# Current Status and Future Prospects of Copper Oxide Heterojunction Solar Cells

**DOI:** 10.3390/ma9040271

**Published:** 2016-04-07

**Authors:** Terence K. S. Wong, Siarhei Zhuk, Saeid Masudy-Panah, Goutam K. Dalapati

**Affiliations:** 1 NOVITAS, School of Electrical and Electronic Engineering, Block S2, Nanyang Technological University, Nanyang Avenue, Singapore 639798, Singapore; ZHUK0003@e.ntu.edu.sg; 2 Institute of Materials Research and Engineering, A*STAR (Agency for Science, Technology and Research), 2 Fusionopolis Way, #08-03, Innovis 138634, Singapore; SAEID1@e.ntu.edu.sg

**Keywords:** cuprous oxide, cupric oxide, heterojunction, solar cell, oxidation, magnetron sputtering, pulsed laser deposition

## Abstract

The current state of thin film heterojunction solar cells based on cuprous oxide (Cu_2_O), cupric oxide (CuO) and copper (III) oxide (Cu_4_O_3_) is reviewed. These p-type semiconducting oxides prepared by Cu oxidation, sputtering or electrochemical deposition are non-toxic, sustainable photovoltaic materials with application potential for solar electricity. However, defects at the copper oxide heterojunction and film quality are still major constraining factors for achieving high power conversion efficiency, η. Amongst the Cu_2_O heterojunction devices, a maximum η of 6.1% has been obtained by using pulsed laser deposition (PLD) of Al*_x_*Ga_1−*x*_O onto thermal Cu_2_O doped with Na. The performance of CuO/n-Si heterojunction solar cells formed by magnetron sputtering of CuO is presently limited by both native oxide and Cu rich copper oxide layers at the heterointerface. These interfacial layers can be reduced by using a two-step sputtering process. A high η of 2.88% for CuO heterojunction solar cells has been achieved by incorporation of mixed phase CuO/Cu_2_O nanopowder. CuO/Cu_2_O heterojunction solar cells fabricated by electrodeposition and electrochemical doping has a maximum efficiency of 0.64% after surface defect passivation and annealing. Finally, early stage study of Cu_4_O_3_/GaN deposited on sapphire substrate has shown a photovoltaic effect and an η of ~10^−2^%.

## 1. Introduction

**Figure 1:**
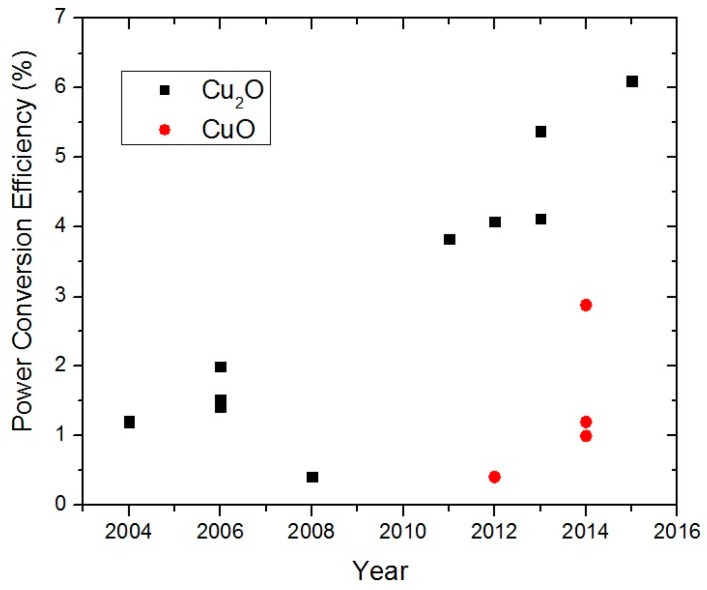
Reported power conversion efficiency of Cu_2_O and CuO heterojunction solar cells *vs.* publication year. For Cu_2_O, the efficiency for 2008 refers to a device prepared by an electrodeposition method instead of the PLD method [8].

When oxidized, copper can form three types of oxides, namely: cuprous oxide (Cu_2_O, cuprite), cupric oxide (CuO, tenorite) and Cu_4_O_3_ (paramelaconite) [1]. All three forms of copper oxides are semiconductors, and Cu_2_O, in particular, is one of the first semiconductors studied for device applications. In the early 20th century, Cu_2_O Schottky junctions were intensively investigated for use as rectifiers in radio receivers [2]. A review of the research conducted during this period, including the difficulty of n-type doping, was written by Brattain in 1950 [3]. After the invention of the point contact transistor in 1947 and the metal oxide semiconductor field-effect transistor in 1960, interest in Cu_2_O declined because of the availability of single crystal silicon and germanium, which can be doped n- and p-type [2]. During the 1970s, there was a resurgence of interest in Cu_2_O as a semiconductor for photovoltaics (PV) because of the need to develop terrestrial PV devices for solar energy conversion in response to the first oil crisis of 1973. The Cu_2_O solar cells investigated during this period were Schottky junction PV devices [4,5,6]. Two research projects on these devices supported by the U.S. National Science Foundation were carried out at the Joint Center for Graduate Study [5,6]. This focus on the Schottky junction structure was due to the difficulty of forming p-n homojunctions in Cu_2_O. Owing to chemical reduction of the Cu_2_O surface to Cu during sputter deposition, the best η obtained was only of the order of 1% regardless of the metal deposited [6]. Nevertheless, it was recognized that a heterojunction or metal-inuslator-semiconductor structure should improve the performance of Cu_2_O PV devices. The low η that could be achieved in these Cu_2_O PV devices eventually led to another waning of interest by the 1980s. A review of the literature on Cu_2_O Schottky junction devices during this period can be found in [7]. Despite this, research on Cu_2_O PV devices continued in Japan, Italy and elsewhere after the 1990s. The Cu_2_O PV research in this recent period is concerned with a search for newer dopants for Cu_2_O, suitable transparent n-type semiconductors for heterojunction formation and low damage junction formation techniques. As a result, there has been significant improvements in the reported η of Cu_2_O PV devices. This can be seen from Figure 1, which is based on a compilation of published η values in the PV literature [8]. In 2015, an η value of 6% was reported for a heterojunction PV device based on Cu_2_O and another n-type semiconductor. This value is exactly triple that of the best η value reported in 2006 for a Cu_2_O heterojunction PV device (see Section 3). Figure 1 shows that, in addition to Cu_2_O, there has been growing research interest in CuO PV devices in recent years. The highest reported η as of 2015 for a CuO based PV device is 1.2%.

It is useful to first consider why an old semiconductor such as copper oxide still attracts such a niche interest at a time when many new semiconductors such as the perovskite and organic semiconductors are being studied for PV applications. In the latest solar cell efficiency tables (version 47) [9], there is no listing for copper oxide devices. Textbooks on PV devices usually have no coverage of copper oxide PV devices [10,11]. The main exception is the reference by Fonash, which has brief mention of Cu_2_O [12]. Sustainability is the main reason for the sustained interest in the copper oxides for PV. Both copper and oxygen are abundant elements like silicon and thus there can be no supply concern in the long run [13]. This is one of the four requirements for a semiconductor to be usable for large scale PV power generation [14]. As discussed extensively in [15,16], a careful sustainability analysis of a large number of semiconductor materials showed that Cu_2_O and CuO are sustainable PV materials. In addition, all three oxides of copper are non-toxic and can be deposited as thin films relatively simply at low cost. The copper oxides are p-type oxide semiconductors uniquely suitable for PV applications. Although there are a few alternative p-type binary oxides such as tin monoxide (SnO) and nickel oxide (NiO), their energy band gaps of 2.5 eV and 3.6–4.0 eV, respectively, are too wide for PV applications [17]. Hence, at present, these transition metal oxides are mainly used for transparent semiconductors.

This review will focus on recent developments in copper oxide heterojunction solar cells and complements the earlier review by Rakhshani [7]. It is organized into seven sections. In Section 2, we first outline the theory of heterojunction solar cells with emphasis on the copper oxide heterojunction and band diagram. Section 3 and Section 4 review the Cu_2_O and CuO heterojunction solar cells, respectively. Section 5 describes the electrodeposited Cu_2_O/CuO heterojunction and its PV properties. Section 6 will briefly discuss preliminary studies on solar cells based on Cu_4_O_3_. This is followed by a conclusion and outlook on the remaining challenges in the field of copper oxide solar cells.

## 2. Heterojunction Solar Cell Device Physics

**Figure 2:**
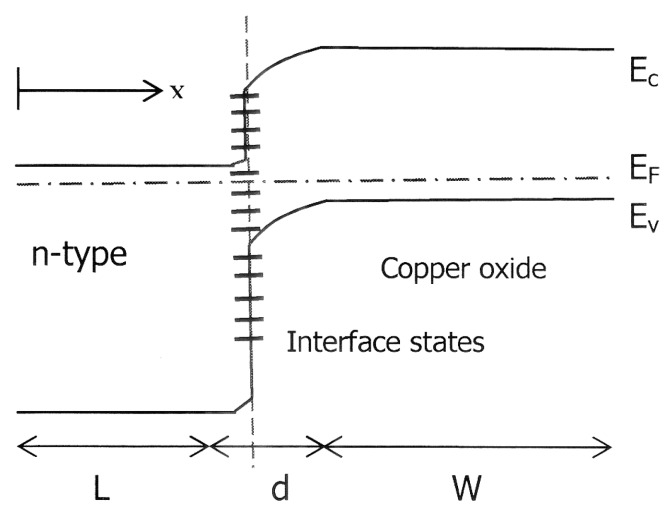
Schematic energy band diagram for heterojunction solar cell of copper oxide and an n-type semiconductor. Interface states are represented by **-** .

Before reviewing the copper oxide heterojunction solar cells, it is useful to consider the energy band diagram and the theory of heterojunction solar cells. Cu_2_O and CuO form type II heterojunctions with another semiconductor such as ZnO or Si in these solar cells. In a type II (or staggered) heterojunction, only one of the band edges (either *E*_c_ or *E*_v_) of one semiconductor is situated within the band gap of the other semiconductor forming the heterojunction [12]. Figure 2 illustrates the schematic energy band diagram of a copper oxide n-type semiconductor heterojunction with interface defects to be discussed in the next section. The n-type layer has the wider energy gap in this heterojunction and therefore sun light is incident through this window layer to reach the Cu_2_O substrate. There is band bending on both sides of this junction and the built-in electric field separates the photo-generated electrons and holes as in a homojunction solar cell. By applying the one-dimensional theory of the semiconductor-semiconductor heterojunction cell in [12], the current density *J* from the device under illumination can be written as:

(1) $$J=-q[\int_{0}^{L+d+W}\int_{\lambda }G_{ph}\left(\lambda ,x\right)d\lambda dx-\int_{0}^{L+d+W}R\left(x\right)dx-J_{ST}\left(0\right)-J_{SB}\left(L+d+W\right)-J_{IR}].$$

Here, *q* is the electronic charge; *d* is the thickness of the neutral p-type layer; *W* is the depletion layer width and *L* is the thickness of the neutral n-type layer. The free carrier photogeneration function, *G_ph_* is integrated with respect to wavelength over the full spectrum of incident sunlight and over the thickness of the solar cell. *R* represents the rate of recombination in the neutral bulk regions of the p- and n-type regions of the heterojunction. *J*_ST_ and *J*_SB_ are the reductions in current density due to recombination in the top and bottom contacts, respectively. The last term *J*_IR_ on the right hand side of Equation (1) is unique to heterojunction solar cells and accounts for recombination at the heterojunction interface. Although another closed form analytical expression for the current density of the heterojunction solar cell exists [18], the equation given above especially shows clearly the effects of recombination on the current density obtainable from the heterojunction solar cell.

The rate of recombination is characterized by the minority carrier lifetime *τ_n_*. For a p-type semiconductor with dopant density *N*_A_, *τ_n_* is given by:

(2) $$\tau_{n}^{-1}=BN_{A}.$$

Here, *B* is the radiative recombination coefficient [19]:

(3) $$B=\frac{{\left(2\pi \right)}^{1/2}hq^{2}}{3{\left(m^{2}c^{3}\right)}^{2}{\left(kT\right)}^{3/2}}{\left\{\frac{m}{m_{e}+m_{h}}\right\}}^{3/2}\left\{1+\frac{m}{m_{e}}+\frac{m}{m_{h}}\right\}nE_{g}^{2},$$

where *h* is the Planck constant; *k* is the Boltzmann constant; *T* is the absolute temperature and *c* is the speed of light. *m* is the free electron mass and *m*_e_ and *m*_h_ are the electron and hole effective mass, respectively. *n* is the refractive index and *E*_g_ is the energy band gap of the semiconductor. The quadratic dependence of *B* on *E*_g_ shows that a semiconductor with a narrower band gap will have a longer minority carrier lifetime. On the other hand, a lower dopant density can also increase *τ_n_*.

In addition to the reduction in current density, the main effect of carrier recombination within a heterojunction solar cell is a decrease in the open circuit voltage, *V*_oc_. For a p-type layer thickness of *w* and low level excitation conditions, *V*_oc_ is given by [20]:

(4) $$V_{\text{oc}}=\frac{E_{g}}{q}-\frac{kT}{q}ln(\frac{qwBN_{c}N_{v}}{J_{\text{ph}}}),$$

where *N*_c_ and *N*_v_ are the effective density of states for the conduction band and valence band, respectively, and *J*_ph_ is the photocurrent density. The second term of this equation shows that for both increased *B* and reduced *J*_ph_, the magnitude of *V*_oc_ is reduced. This shows the crucial impact of recombination on the photovoltaic performance of heterojunction solar cells.

## 3. Cu_2_O Thin Film Heterojunction Solar Cells

Cu_2_O can be formed by thermal oxidation of high purity Cu foils in a furnace [21]. The material properties of Cu_2_O have been studied in detail and can be found in other reviews [1]. Here, for the sake of completeness, only a summary of those properties of Cu_2_O relevant to solar cells will be described. Cu_2_O has a cubic unit cell structure and an energy band gap of 1.7–2 eV depending on the deposition conditions [1]. Since the band gap of Cu_2_O is direct, the absorption coefficient of Cu_2_O is relatively high (~10^5^ cm^−1^) and is comparable to some organic semiconductors. As-deposited undoped Cu_2_O is a p-type semiconductor with a majority carrier mobility of ~100 cm^2^/V∙s. The p-type conductivity is due to Cu vacancies in the Cu_2_O [1]. For extrinsic doping, N and Cl are known to be p-type dopants for Cu_2_O [22]. The search for n-type dopants for Cu_2_O has until recently been elusive [3]. However, as will be discussed in Section 4 and Section 5, respectively, methods for preparing n-type CuO_x_ and Cu_2_O are now available.

**Figure 3:**
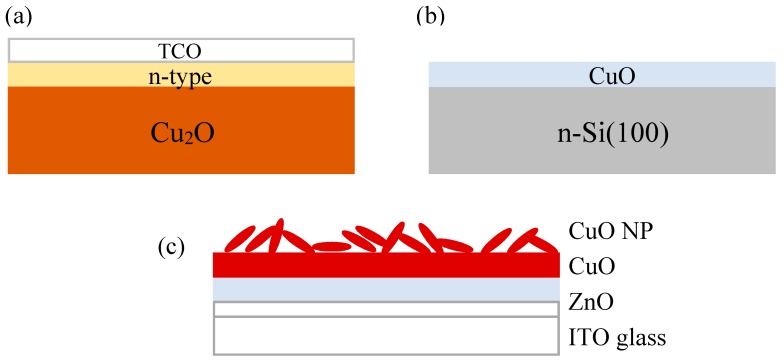
Schematic device structure for (**a**) Cu_2_O/ZnO heterojunction solar cell; (**b**) CuO/n-Si heterojunction solar cell; and (**c**) CuO/ZnO heterojunction solar cell enhanced by mixed phase CuO/Cu_2_O nanopowder.

Cu_2_O heterojunction solar cells have a device structure similar to other thin film solar cells (Figure 3a). The Cu_2_O is both the absorber and a substrate upon which is deposited a thin layer of n-type wide band gap semiconductor. This layer may have a third transparent conducting oxide (TCO) layer deposited on top of it. Ohmic contacts are formed to both the top and bottom of the device. The first published report of Cu_2_O heterojunction solar cells with this structure was that by Herion *et al.* in 1980 [23]. These investigators used the sputtering technique to deposit ZnO onto Cu_2_O sheets. The PV characteristics of this device is tabulated in Table 1 (see Section 5) together with other key devices discussed in this review. Although an η of 0.14% was measured for the ZnO-Cu_2_O device, the photovoltaic effect was not due to a ZnO-Cu_2_O heterojunction. Rather, it was due to the Schottky barrier between a thin Cu film formed by reduction of the Cu_2_O surface during the ZnO sputter deposition. This paper highlights the susceptibility of the Cu_2_O surface to solid state reaction and hence the need to choose a low energy, low damage deposition method to form a true semiconductor heterojunction with Cu_2_O.

A more recent example of a thin film p-Cu_2_O/n-ZnO heterojunction solar cell fabricated on glass substrates by sputtering is the work of Akimoto *et al.* [24]. In this study, the Cu_2_O layer was deposited by reactive radio frequency (rf) magnetron sputtering using a Cu target while the ZnO layer was deposited by magnetron sputtering. The sequence of deposition was found to have a major influence on the photovoltaic properties of the heterojunction fabrication. For the glass/Au/p-Cu_2_O/i-ZnO/n-ZnO (Cu_2_O first) structure, the reverse leakage current was relatively high and a photovoltaic effect was almost unobservable [24]. On the other hand, the glass/n-ZnO/i-ZnO/p-Cu_2_O (ZnO first) structure showed much better rectification and an η of 0.4% was obtained [24]. The difference between the two device structures was attributed to the similar atomic arrangements in the crystal structures of ZnO and the Cu_2_O for the ZnO first structure, which results in fewer interface defects. However, both *V*_oc_ and short circuit current density *J*_sc_ are low because of these defects. 

In order to achieve η above 1%, it is necessary to identify suitable n-type semiconductors that can be deposited onto Cu_2_O [25]. This is because solar cells fabricated from Cu_2_O substrates tend to have higher efficiency than cells made from Cu_2_O thin films [7]. In one such study [26], Tanaka *et al.* deposited several transparent conducting oxides (TCO) thin films, namely In_2_O_3_, In_2_O_3_:Sn (ITO), ZnO, ZnO:Al (AZO) and ZnO-In_2_O_3_ by PLD onto Cu_2_O sheets. Both In_2_O_3_ and ITO gave poorly rectifying heterojunctions and poor PV performance. ZnO-Cu_2_O formed rectifying junctions and the current voltage characteristics both in the dark and under illumination were found to be affected by the O_2_ pressure during deposition [26]. The highest η obtained for ZnO-Cu_2_O was 0.9% under illumination by air mass AM 2 solar spectrum. On the other hand, an η of over 1% can be achieved by using the AZO-Cu_2_O heterojunction. This is because AZO has a smaller work function than ZnO [26]. For AZO, the critical parameter is the deposition temperature during PLD. By controlling this temperature to the range of 150 °C–200 °C, a rectifying junction and an η of 1.2% under AM2 illumination was obtained. The improvement in η of the AZO-Cu_2_O junction is attributed to a broader spectral response that results in higher *J*_sc_ [26]. The results of refs [25] and [26] show that ZnO and AZO are suitable n-type semiconductors for forming heterojunctions with Cu_2_O.

Until 2006, the η of Cu_2_O heterojunction solar cells reported in the literature remained under 2%. Since this is obviously inferior to competing second generation semiconductor solar technologies and much lower than the theoretical limit of Cu_2_O devices of 20% for the AM1 spectrum [5], systematic studies were carried out to improve the fabrication technique of the Cu_2_O cells. In one such study, Minami *et al.* investigated four techniques for depositing the n-type thin film semiconductor on a Cu_2_O absorber substrate [27]. These include: (i) DC magnetron sputtering; (ii) rf magnetron sputtering; (iii) PLD and (iv) vacuum arc plasma evaporation (VAPE). The VAPE technique involves the use of a low voltage discharge to generate an arc plasma to evaporate the source material [28]. AZO was deposited by DC and RF magnetron sputtering at different substrate temperatures and for different sample orientation and positions in the sputtering system. The film properties of the AZO such as crystallinity, carrier concentration, Hall mobility and resistivity were all strongly dependent on the substrate temperature and sample orientation. Since the properties of the AZO film affect the properties of the AZO/Cu_2_O junction, the photovoltaic parameters show a similar dependence on deposition conditions. It was concluded that at the optimum substrate temperature and for a sample normal to the target, the improved PV performance was a result of fewer defects at the heterojunction and an improved crystallinity of the AZO [27]. This in turn suggests that magnetron sputtering, DC or RF, may not be the best deposition technique for forming the AZO/Cu_2_O heterojunction. PLD and VAPE were therefore used to deposit ZnO on Cu_2_O in addition to DC and RF magnetron sputtering. Both PLD and VAPE do not involve ion bombardment of the Cu_2_O surface which can generate electronic defects. Furthermore, the absence of reactive oxygen species preclude the oxidation of the Cu_2_O surface [27]. As a result, the η of ZnO/Cu_2_O devices fabricated by PLD (1.42%) and by VAPE (1.52%) were both higher than devices fabricated by magnetron sputtering [27]. This is the reason why the highest performance Cu_2_O heterojunction devices are usually fabricated by PLD nowadays. 

**Figure 4:**
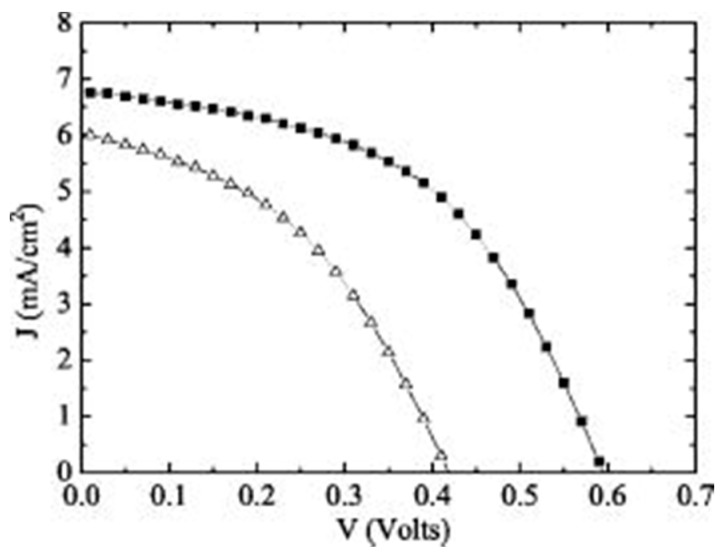
Current density-voltage characteristics of Au/Cu_2_O/ZnO/ITO/MgF_2_ device (upper) and Au/Cu_2_O/ZnO/MgF_2_ device (lower) under AM 1.5G illumination Reprinted from Applied Physics Letters 88, 163502 (2006) with the permission of AIP Publishing [29].

A Cu_2_O heterojunction solar cell with an η above 2% was reported by Mittiga *et al.* in 2006 [29]. This device has a four layer structure and consists of a thermally oxidized Cu_2_O substrate, a ZnO n-type layer, an indium doped tin oxide (ITO) window layer and an antireflection MgF_2_ layer. The oxidation conditions used for the Cu_2_O layer resulted in a polycrystalline structure with large grain size and high hole mobility. A low resistivity (~1 kΩm) was realized by quenching the Cu_2_O sheets after cooling to 450 °C in a furnace [29]. The ZnO layer was deposited onto the Cu_2_O substrate by ion beam sputtering (IBS) followed by a thicker layer of ITO deposited by the same technique. Under AM 1.5G (global) illumination, the Au/Cu_2_O/ZnO/ITO/MgF_2_ device has a measured η of 2.01% [29]. This was higher than the reference Au/Cu_2_O/ITO/MgF_2_ device as shown by the *J-V* characteristics in Figure 4. The *V*_oc_ values for both devices were considered to be influenced by defects at the heterointerface. These cause reduction in the shunt resistance and increased the reverse saturation current. As a result, improvement of interface defects should increase the *V*_oc_ and η.

The first report of a Cu_2_O heterojunction solar cell with an η above 3% was reported by the Kanazawa Institute of Technology in 2011. In the first of a series of papers [30,31,32,33], Minami and co-workers demonstrated an improved device architecture for Cu_2_O devices. In this structure, a polycrystalline Cu_2_O substrate and absorber was first formed from Cu by using a three step oxidation process [30]. Subsequently, an n-type layer of undoped ZnO and a transparent conductor layer of AZO were deposited sequentially by PLD. The ZnO forms a heterojunction with Cu_2_O and the AZO acts as a window layer. The back contact consists of an ohmic contact formed from either Au or Cu_2_S. The photovoltaic parameters *V*_oc_, *J*_sc_, fill factor *FF* and η were studied as a function of the ZnO layer thickness. For a ZnO thickness in the range of 30–50 nm, the η reached a maximum of 3.83% [30]. This was mainly due to improvement in ZnO film properties such as fewer interface defects and reduced resistivity. For a ZnO layer thicker than 50 nm, both *V*_oc_ and FF decreased because of a short minority carrier lifetime [30]. By comparison, the η of an AZO/Cu_2_O reference device of ~1.6% was lower than the optimized AZO/ZnO/Cu_2_O device. This shows that the ZnO buffer layer forms a better quality junction with Cu_2_O in comparison with AZO. However, both the deposition condition and thickness of the ZnO layer must be carefully controlled to obtain high efficiency.

A more in-depth study of the n^+^AZO/ZnO/Cu_2_O device structure was carried out by Nishi *et al.* [31]. The device fabrication process was similar to that in [30]. The thickness of the ZnO buffer layer was varied between 0 nm and 150 nm where a zero ZnO thickness corresponded to an n^+^AZO/Cu_2_O Schottky barrier junction. At the optimum ZnO thickness of 50 nm, an η of 4.08% was measured. Dark current voltage measurements showed that the inserted ZnO buffer layer forms a heterojunction with the Cu_2_O with a built in potential (or barrier height) that is greater than that of the Schottky junction. The increased barrier height could be related to the higher quality interface obtained when ZnO is deposited at room temperature conditions [31]. In addition, the lower carrier concentration of the undoped ZnO layer results in a finite depletion width in the ZnO that increases the total active layer thickness. This is shown by photovoltage spectral response measurement. When compared with the n^+^AZO/Cu_2_O device, the n^+^AZO/ZnO/Cu_2_O device has a broader photovoltage spectrum below 460 nm because of the heterojunction formation [31]. 

**Figure 5:**
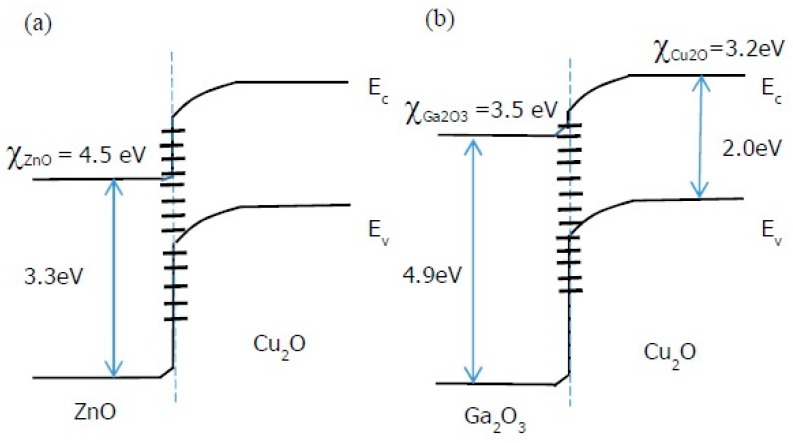
Schematic energy band diagram of (**a**) ZnO/Cu_2_O and (**b**) Ga_2_O_3_/Cu_2_O heterojunction showing difference in conduction band offset. Interface states are represented by **-** .

A still higher η was obtained by Minami *et al.* when the undoped ZnO layer was substituted by undoped n-type Ga_2_O_3_ in an AZO/Ga_2_O_3_/Cu_2_O/Au device structure [32]. The choice of Ga_2_O_3_ was motivated by the fact that the photovoltaic properties of a Cu_2_O heterojunction cell are determined by the energetics of the heterointerface. For the Ga_2_O_3_/Cu_2_O heterojunction, the conduction band offset (Δ*E*_c_) is smaller because the electron affinity *χ* of Ga_2_O_3_ is in between that of Cu_2_O and ZnO (Figure 5) [32,33]. This smaller Δ*E*_c_ should lead to a higher device efficiency. The Ga_2_O_3_ layer in this device was also deposited by PLD using Ga_2_O_3_ pellets in the presence of oxygen. The η and other photovoltaic properties were found to be dependent on the oxygen pressure during PLD and the thickness of the Ga_2_O_3_ layer. At optimum conditions, an η of 5.38% was obtained. The photovoltaic parameters (*V*_oc_, *J*_sc_, FF) were better than those of a comparable AZO/ZnO/Cu_2_O/Au device. The high *V*_oc_ (=0.8 V) was attributed to reduced carrier recombination due to reduced defects at the heterointerface. This was demonstrated using dark current voltage and external quantum efficiency measurements. 

The Cu_2_O heterojunction solar cell with the highest η (as of 2015) was reported by Minami *et al.* [34]. This cell was fabricated on a Cu_2_O substrate formed by thermal oxidation of high purity Cu sheets. The Cu_2_O sheet serves as both the solar absorber and the device substrate. In order to reduce the series resistance of the heterojunction solar cell, the resistivity of the Cu_2_O substrate was reduced by Na doping [34]. This was carried out by heating the substrate with NaI in an inert atmosphere at 500 °C–1000 °C [34]. The reason for doping by Na is that, unlike other dopants, the Cu_2_O resistivity decrease is not accompanied by a decrease in the Hall mobility for Na dopants. From their experiments, an optimum resistivity of the Cu_2_O:Na was found to be ~15 Ωcm. The heterojunction was formed by successive ArF excimer laser PLD of a layer of n-type aluminum gallium oxide (Al*_x_*-Ga_1-*x*_-O) and a top window layer of AZO. The photovoltaic performance of the device was found to depend strongly on the composition of the Al*_x_*Ga_1-*x*_O. At an optimized Al concentration of 2.5 at% (*i.e.*, Al_0.025_-Ga_0.975_-O), the highest η of 5.72% was achieved [34]. This was increased further to 6.1% with the use of MgF_2_ antireflection layer and an optimized Al_0.025_-Ga_0.975_-O layer thickness [34].

The chronological discussion above shows that substantial progress has indeed been accomplished in recent years in polycrystalline Cu_2_O heterojunction solar cells. This advancement can be attributed to four key developments. The first is the improvement in the thermal oxidation of Cu resulting in Cu_2_O with improved crystallinity. The second is the use of thin buffer layers and CuO etching prior to n-type semiconductor deposition. This results in an improved interfacial layer with reduced defects. Third, the synthesis of n-type oxide semiconductors with tailored band edge offset with respect to Cu_2_O can enhance junction properties. Finally, the use of PLD for n-type oxide semiconductor deposition crucially improves the heterojunction interface. However, the PLD process is difficult to be scaled up to large area substrates. This is because the source material is ablated from a point on the target. In this context, we briefly highlight two related process developments. 

The first is the photo-metalorganic chemical vapor deposition (MOCVD) of Cu_2_O by Gupta *et al.* [13]. In this photo-MOCVD process, an organometallic precursor containing Cu carried by ultra high purity gases into a deposition chamber is used for Cu_2_O deposition onto p-Si substrates. The deposition at 750 °C is assisted by ultraviolet photons from a Xenon flash lamp. Although structural, optical and electrical characterization of these Cu_2_O films on p-Si and quartz were performed, no data on PV devices was reported [13].

**Figure 6:**
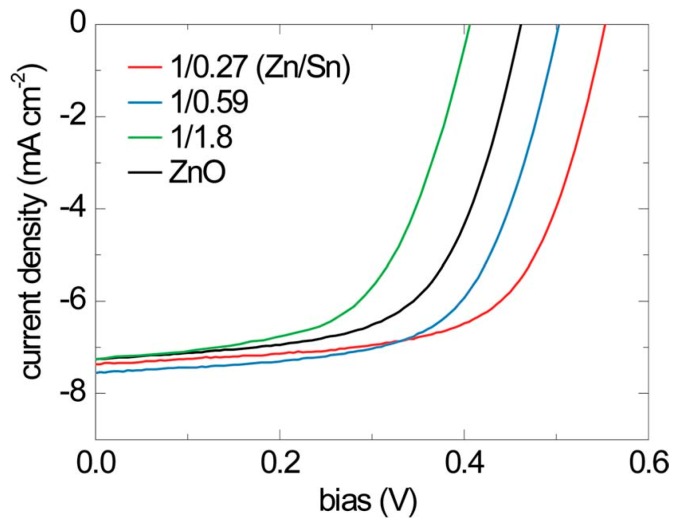
Current density-voltage characteristics of ZnO:Al/a-ZTO/Cu_2_O devices with different composition of ALD buffer layer and no buffer layer under AM 1.5G illumination. *Energy Environ. Sci.* 2013, **6**, 2112—Published by The Royal Society of Chemistry [35].

The second concerns the use of atomic layer deposition (ALD) to control the interface quality of Cu_2_O heterojunction soloar cells. In a study by Lee *et al.* [35], an ultra thin buffer layer of amorphous zinc tin oxide (a-ZTO) was deposited by ALD onto electrochemically deposited Cu_2_O. a-ZTO reduces recombination at the interface by acting as an electron blocking layer. The *J-V* characteristics for three ZnO:Al/a-ZTS/Cu_2_O/Au devices with different Zn:Sn cationic ratios in a-ZTS is shown in Figure 6. At optimized composition of a-ZTO, an η of 2.65% was measured for these devices which is higher than the control device without a-ZTO. In a subsequent study [36], it was further demonstrated that the ALD reaction conditions can be used to control the oxidation state of Cu and prevent the formation of an undesirable CuO interfacial layer. When the ALD temperature of the a-ZTO layer was decreased, the *V*_oc_ of ZnO:Al/a-ZTS/Cu_2_O/Au devices increased, indicating reduced defects at the a-ZTS/Cu_2_O interface. The highest η of 3.06% was observed at a deposition temperature of 70 °C. This group also demonstrated that insertion of 20 nm thick extrinsic Cu_2_O:N between the Cu_2_O and Au layers can effectively reduce contact resistance of this device structure and improve FF. This is because Cu_2_O:N acts as a p-type transport layer and facilitates hole transport by tunneling [37]. It should be noted that Cu_2_O films have also been used as hole transport layers in perovskite solar cells [38].

## 4. CuO Thin Film Heterojunction Solar Cells

Relatively little is known about the material properties of CuO. CuO has a monoclinic crystal structure. It has a direct energy band gap of ~1.5 eV and the absorption coefficient is also high [1]. Since for a single p-n junction solar cell, the optimum band gap is 1.4 eV, the optical properties of CuO make it a very suitable semiconductor absorber material for solar cell applications [11]. In addition, the electrical properties of CuO such as majority carrier mobility and minority carrier diffusion length are also adequate. 

Although the narrower band gap of CuO is a better match to the solar spectrum compared with Cu_2_O, little research on CuO heterojunction solar cells has been published until recently [39,40,41,42,43,44]. The structure of the CuO heterojunction solar cells reported so far typically involves deposition of the CuO thin films onto a semiconductor substrate such as crystalline Si wafers (Figure 3b). The thermal oxidation method widely used for Cu_2_O devices has not been used. In 2012, Gao *et al.* reported a p-CuO/n-Si heterojunction solar cell fabricated by the reactive magnetron sputtering technique [39]. The CuO layer was deposited by using a Cu target and a sputtering plasma consisting of Ar and O_2_. When the flow rate ratio of O_2_ to Ar was optimized, the deposited CuO has a nanocrystalline structure with a crystallite size of ~8 nm [39]. The optical band gap of the CuO layer was 1.07 eV and the hole mobility deduced from Hall measurements was ~0.15 cm^2^/V∙s [39]. Under AM 1.5G illumination, the current density voltage (*J-V*) characteristic showed a modest photovoltaic effect and an η of 0.41% was measured for a Cu/p-CuO/n-Si/Al device structure [39]. Both *V*_oc_ and FF were low because the high series resistance of the nanocrystalline CuO layer and defects at the p-CuO/n-Si interface resulted in carrier recombination. The very thin CuO layer (100 nm) and the Cu grid top electrode might have also limited the amount of light absorbed by this device structure.

**Figure 7:**
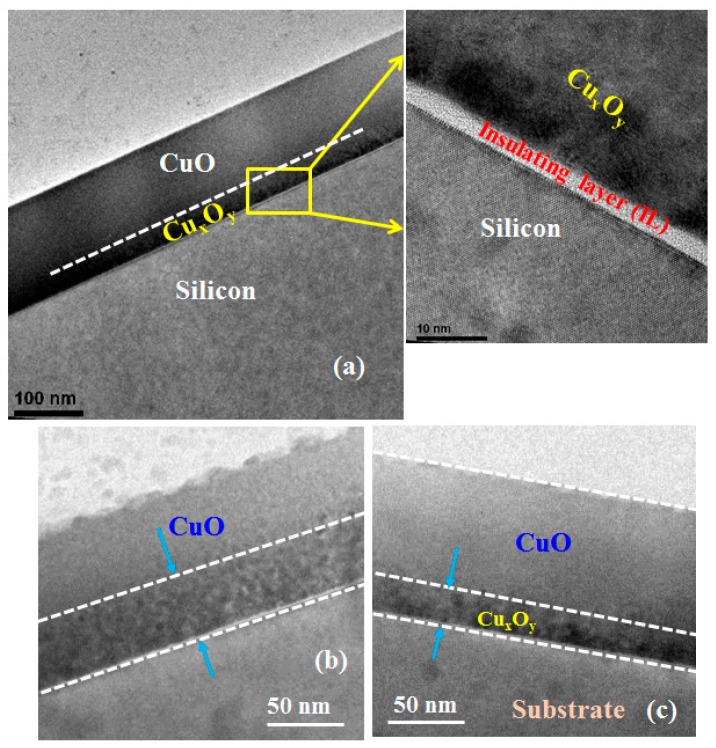
Cross-sectional TEM of p-CuO/n-Si heterostructure. Sputter deposited CuO at 150 W on n-Si substrate for (**a**) as-deposited; and (**b**) after thermal treatment at 300 °C for 1 min; (**c**) CuO deposited at 50 W sputtering power and annealed at 300 °C for 1 min.

Another early study of the CuO/Si heterojunction is that by Kumar *et al.* [40]. These investigators used the RF argon sputtering of a CuO target to deposit CuO onto n-Si(100). After deposition, the polycrystalline CuO films were annealed in nitrogen at 300 °C. The crystalline structure of the film was determined by X-ray diffraction to be predominantly CuO phase for an annealing temperature of 300 °C [40]. Under dark conditions, the CuO/Si heterojunction showed a rectifying *J-V* characteristic with a rectification ratio of 10^4^ (at +3 V and −3 V) [40]. When illuminated, a photovoltage response was observed [40]. It is also worth noting that there is a presence of interfacial oxide layer between the silicon substrate and p-CuO layer. High resolution transmission electron microscope (HRTEM) analysis reveals that the thickness of the interfacial layer is around 4 nm. Furthermore, a Cu-rich interfacial layer between p-CuO and silicon substrate is also observed and the Cu-rich interface layer significantly depends on the sputtering power, as shown in Figure 7.

**Figure 8:**
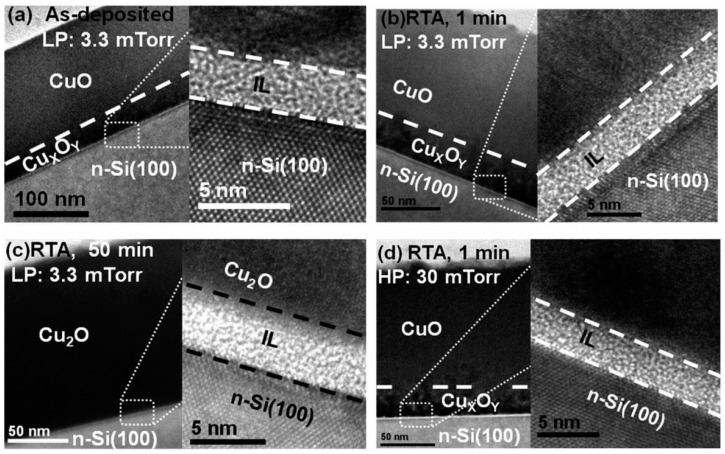
Cross sectional TEM image of 150 nm thick CuO films deposited on n-type Si(100) substrates at a working pressure of 3.3 mTorr: (**a**) as-deposited; (**b**) annealed at 300 °C for 1 min; and (**c**) annealed at 300 °C for 50 min. The same thickness CuO film deposited at a working pressure of 30 mTorr and annealed for 1 min at 300 °C is shown in (**d**) © 2014 John Wiley & Sons, Ltd. [41].

A CuO heterojunction solar cell with PCE approaching 1% was reported recently by Masudy-Panah *et al.* [41]. The heterostructure studied was Al/Ti/n-Si/p-CuO/Ti/Al. The CuO layer was deposited by argon RF sputtering of a CuO target at a fixed power of 150 W and working pressure in the range of 3 to 35 mTorr. After deposition, the CuO film was further annealed at 300 °C from 1 min to 1 h. The CuO film properties are influenced strongly by both the working pressure and annealing time. By using X-ray diffraction and Raman spectroscopy, the CuO phase was found to have improved crystallinity when a higher working pressure was used. In addition, when annealed for a prolonged period, there is a phase transformation from CuO to Cu_2_O and a mixture of CuO and Cu_2_O was formed as a result. This phase transformation is corroborated by X-ray photoelectron spectroscopy analysis. The position of the Cu 2p peak shifted from 934.0 eV to 932.4 eV [41]. In the Raman spectrum, the formation of Cu_2_O is signified by the appearance of peaks between 119 cm^−1^ and 500 cm^−1^. In addition, a peak at 1110 cm^−1^ indicates that elemental Cu is present in the CuO as a Cu rich copper oxide. This Cu rich copper oxide within the CuO was observed by HRTEM to be about ~30 nm thick for a CuO film thickness of 150 nm, as shown in Figure 8 [41]. In between the Cu rich copper oxide and the Si substrate is an amorphous interfacial layer (IL) due to residual oxygen in the sputtering system. Its composition was found by energy dispersive X-ray (EDX) spectroscopy to be comprised of Si, Cu and O [41]. 

**Figure 9:**
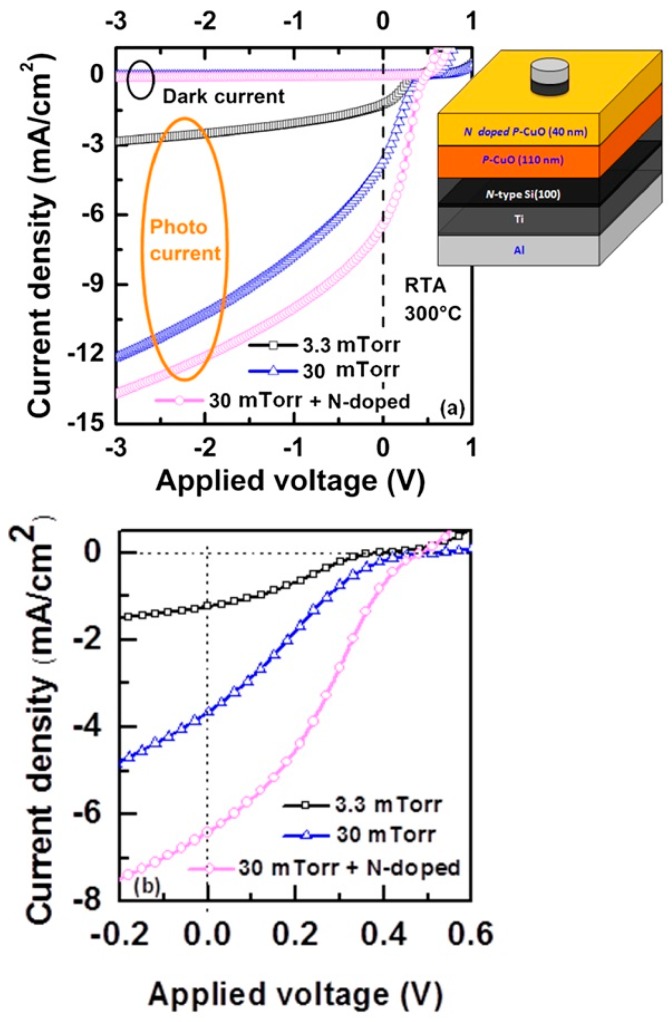
(**a**) Comparison of dark current and photocurrent densities of p-CuO/n-Si heterojunction solar cells with CuO deposited at 3.3 and 30 mTorr working pressures. The open circuit voltage and photocurrent increased significantly for the sample deposited under high working pressure. Inset of the figure shows schematic diagram of nitrogen-doped CuO device; (**b**) Variation of open circuit voltage and short circuit current of the solar cells © 2014 John Wiley & Sons, Ltd. [41].

Both the Cu rich copper oxide and the amorphous IL are detrimental to interface quality and thus need to be minimized by controlling the sputtering conditions of the CuO. This was demonstrated by photocurrent voltage characteristics of Al/Ti/p-CuO/n-Si/Ti/Al devices. The *V*_oc_ of devices with CuO deposited at low working pressure and annealed at 300 °C for 1 min was 380 mV and the η was only 0.14% [41]. When the annealing time was increased, the *V*_oc_ decreased further due to the thickening of the IL and the Cu rich copper oxide layer. On the other hand, the *V*_oc_ of the device increased to 509 mV when the CuO layer was deposited at 30 mTorr and annealed at 300 °C for 1 min [41]. This improvement in *V*_oc_ is due to thinner interfacial layers and better CuO crystallinity. As a result, a higher η of 0.36% was obtained. This study shows that the CuO deposition and annealing conditions play a crucial role in determining the achievable photovoltaic performance of p-CuO/n-Si heterojunction solar cells. Furthermore, through the introduction of highly nitrogen doped CuO thin film in between CuO layer and top metal contact, the fill factor of the device improved significantly and, as a result, efficiency of the device increased to 1%, as shown in Figure 9.

**Figure 10:**
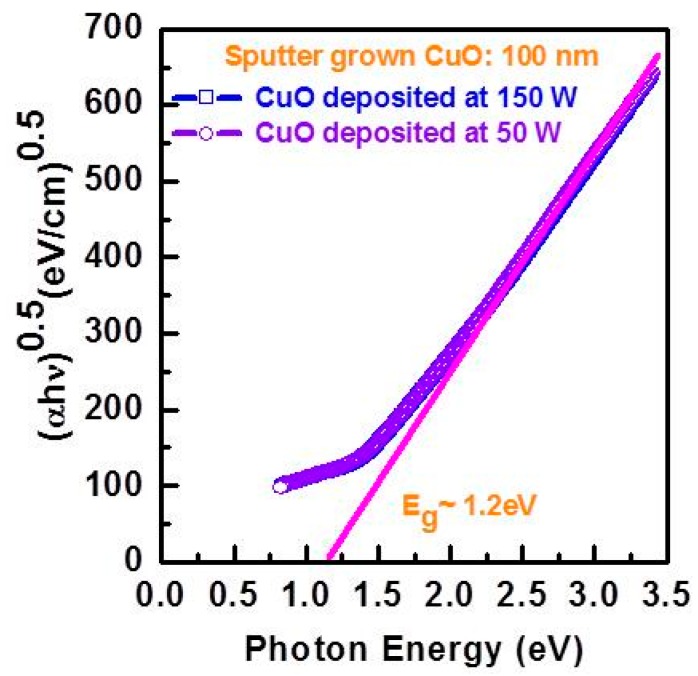
Tauc plot of (*αhν*)^0.5^ *vs*. photon energy of CuO thin film grown at sputtering power of 50 W and 150 W followed by rapid thermal treatment at 300 °C for 1 min. The extrapolated intercept gives the optical band gap of CuO.

**Figure 11:**
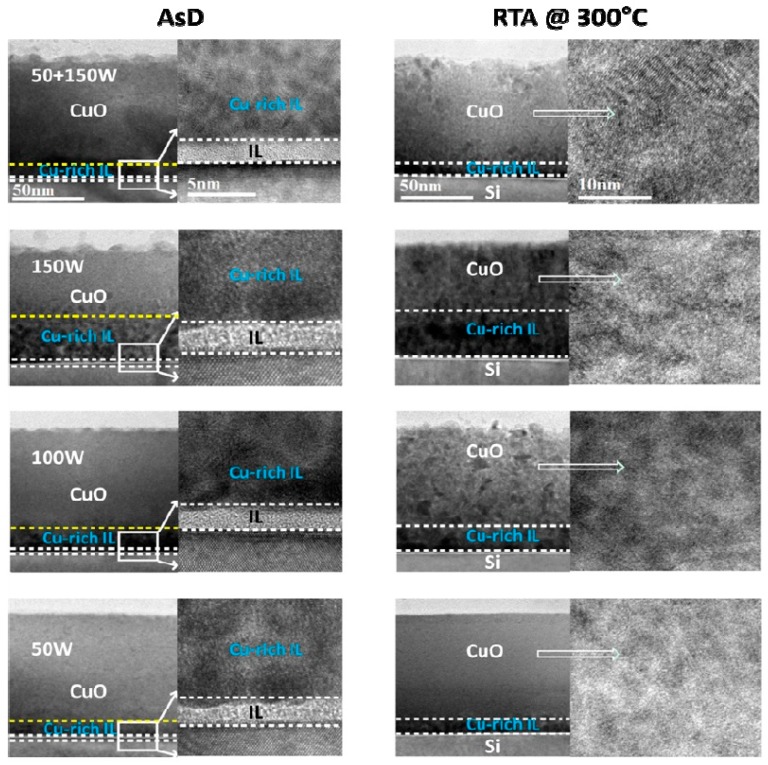
HRTEM image of p-CuO/n-Si hetreojunction. p-CuO was deposited on n-Si using different radio frequency power of 50 W, 100 W, 150 W and by using two-step sputter. There is significant impact of radio frequency power on the material quality and interface properties. Reprinted with permission from Journal of Applied Physics **116**, 074501 (2014) Copyright 2014, AIP Publishing LLC [42].

Since the poor photovoltaic performance of p-CuO/n-Si heterojunction solar cells was attributed to native oxide formation and a Cu rich IL during the sputtering process, a comprehensive study on the IL properties was carried out by Masudy-Panah *et al.* [42]. CuO films were deposited at different RF power (50 W, 100 W and 150 W) onto Si using a stoichiometric CuO target. The composition profile, crystallinity and the structure of the CuO/Si interface was studied by time of flight secondary ion mass spectrometry (TOF-SIMS), Raman spectroscopy, TEM and X-ray diffraction respectively. For an RF power of 50 W, the composition of the CuO layer was quite uniform. When the RF power was increased to 150 W, the Cu rich IL became thicker and the crystalline quality of the CuO improved without compromising the optical band gap of the thin film CuO determined from the Tauc plot of the absorption coefficient *α* and photon energy *hν* (Figure 10). This finding leads to a two-step sputtering process in which a thin layer of CuO is first sputtered at low power (*i.e.*, 50 W) followed by a thicker layer of CuO deposited at 150 W. The resulting CuO can have both good crystallinity and a thin Cu rich IL (Figure 11). In order to demonstrate the improved film properties for the two step process, the photovoltaic performances of several p-CuO/n-Si devices were compared for one-step and two-step deposition of CuO. For the one-step process, the lowest RF power (50 W) led to the highest η of 0.38%. At higher RF power, the increased thickness of the Cu IL alluded to above led to a loss of efficiency. When the two-step process is used instead with the CuO layer deposited at higher power and doped with nitrogen, an η of 1.21% was obtained. This is the first report of a CuO heterojunction solar cell with an η above 1% [42].

Since high sheet resistance and low carrier concentration of a CuO absorber layer are possible reasons of poor photovoltaic performance of copper oxide solar cells, a detailed study on doping of CuO thin films was carried out by Masudy-Panah *et al.* [43,44]. These investigators report that the impact of nitrogen doping on the crystal quality of CuO thin films significantly depends on its concentration and annealing temperature [43]. Thermal treatment of lightly doped samples with N concentration of 1.5% did not cause significant changes in its crystal structure regardless of annealing temperature. However, CuO-Cu_2_O mixed phase was formed for the highly doped samples annealed at low temperatures and a dominant Cu_2_O phase was formed for the highly doped samples annealed at high temperatures.

**Figure 12:**
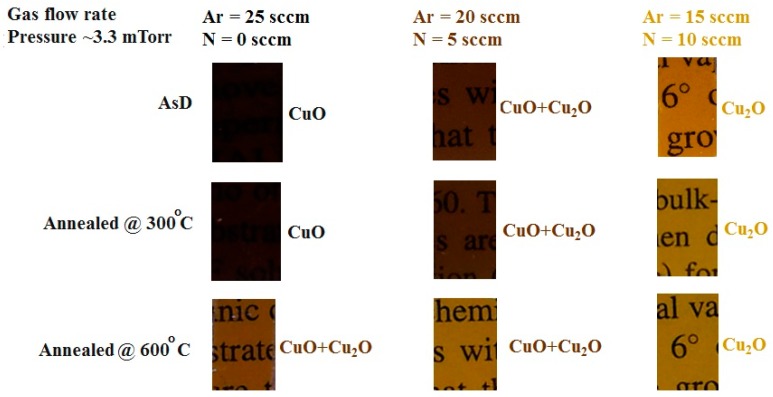
Tuning of film color and optical transmittance through *in situ* nitrogen doping for sputter grown CuO and Cu_2_O thin films. For each deposition condition, thermal annealing modifies film color and transmittance. The wider band gap of Cu_2_O results in higher transmittance as shown by the printed characters beneath these films.

The reflectance and transmittance of N-doped CuO thin films in the wavelength range of 200 nm–1500 nm was found to be increased with increasing nitrogen concentration. It was explained by Masudy-Panah in terms of CuO bandgap widening and the presence of Cu_2_O phase. It was also shown that by tuning the nitrogen concentration during sputter growth of CuO film (*in situ* nitrogen doped), the color of the copper oxide film can be tuned for as-deposited and also for the annealed films as shown in Figure 12. 

Impact of N-doping on photovoltaic properties of p-CuO(N)/n-Si based heterojunction solar cells was also studied [43]. *V*_oc_ of doped samples was found to degrade with increasing N doping concentration. According to the investigators, nitridation of Cu-rich interfacial layer and formation of poor quality copper oxide nitride/Si interface could be the reasons of the *V*_oc_ degradation. Photovoltaic properties of highly doped samples were found to be significantly lower because of CuO bandgap widening and Cu_2_O formation despite of lower sheet resistance. However, lightly doped samples showed better photovoltaic performance over highly doped samples and control samples. Improvements in *J*_sc_, *FF* and η were attributed to the reduction of series resistance of N-doped CuO thin films. 

In another study, Masudy-Panah *et al.* showed that, by Ti-doping, it is possible to significantly reduce resistivity of CuO thin films while maintaining its optical properties and structural quality [44]. The crystal structure of Ti-doped CuO thin films maintained good quality when Ti concentration was increased up to 0.099%. Doping with higher Ti concentrations resulted in formation of secondary defect phases which decreased crystalline ordering. The transmittance and reflectance of Ti-doped CuO were found to be independent of Ti dopant concentration. Furthermore, Ti doping has insignificant impact on surface morphology of CuO films.

The p-CuO(Ti)/n-Si heterojunction solar cells with Ti concentration of 0.099% showed better performance over undoped samples and other Ti-doped samples. Both *V*_oc_ and *J*_sc_ improved in comparison with control samples and other Ti-doped samples. Masudy-Panah *et al.* explained these improvements by reducing of series resistance and enhancing of collection efficiency. When the two-step process was used instead to deposit the Ti-doped CuO absorber layer at high working pressure of 30 mTorr, an η of 1.2% was obtained. However, overall performance of highly doped samples significantly reduced because of high recombination rate with CuO layer.

Dalapati *et al.* reported that CuO thin films deposited by RF magnetron sputtering at high working pressure of 30 mTorr have better crystal quality than the films deposited at lower working pressure of 3.3 mTorr while optical properties remain the same [45]. Investigators showed that CuO thin films grown at high working pressure exhibit lower defect density and higher carrier concentration. 

In the CuO devices discussed thus far, the sputtered CuO layer is always p-type. Recently, however, Lee *et al.* has observed n-type behavior in nanoscale non-stoichiometric CuO_x_ deposited by reactive magnetron sputtering [46]. The n-type conductivity which was confirmed by Hall effect measurements results from the fact that in the initial stage of deposition, the CuO*_x_* is Cu rich and this causes electrons to be majority carriers. By depositing 50 nm of n-CuO_x_ onto intrinsic i-a-Si:H in a heterojunction p-i-n device structure, a maximum η of 3.04% was measured [46]. This can be further increased to 4.79% by inserting a thin n-mc Si:H depletion assisting layer (DAL) between the CuO*_x_* and i-a-Si:H layer. The DAL enhances η of the device by increasing *V*_oc_.

The performance of CuO heterojunction solar cells can be further enhanced by the use of mixed phase copper oxide nanopowders (NP). This innovative approach to boost light absorption was demonstrated by Bhaumik *et al.* in 2014 [20]. The device structure studied consists of glass/ITO/ZnO/CuO/NP(Cu_2_O, CuO) (Figure 3c). This structure is different from all the device structures discussed thus far because sunlight is incident from the transparent substrate rather than from the top of the device. In addition, both ZnO and CuO layers were deposited by PLD from the respective targets using a krypton fluoride (KrF) excimer laser to minimize the defect density at the interface. The mixed phase NP containing both cupric oxide and cuprous oxide was synthesized from CuSO_4_ and KOH by a hydrothermal process within a sealed autoclave at 80 °C. After synthesis, the mixed phase NP dispersed in toluene was drop cast onto the surface of the CuO thin film and annealed in oxygen for up to two hours. For the device annealed in this manner for two hours, the *J*_sc_ under AM1.5 solar illumination was 20.9 mA/cm^2^ [20]. This is much higher than the *J*_sc_ of the control device with only the thin film layers (290 μA/cm^2^) and another device with commercial copper oxide micro-powder (4.22 mA/cm^2^). Interestingly, the *V*_oc_ of the device was also improved by the NP and annealing. After annealing for 2 h, the *V*_oc_ increased from 0.3 V to 0.4 V. As a result, a high η of 2.88% was achieved. This is the highest reported efficiency for a CuO heterostructure solar device. The high performance was explained in terms of: (i) increased light absorption caused by the copper oxide NP; and (ii) improved diffusion of electrons and holes due to removal of ligands from the NP after annealing. As for *V*_oc_, the increase was attributed to the increase in band gap and improved band alignment for the copper oxide layer.

**Figure 13:**
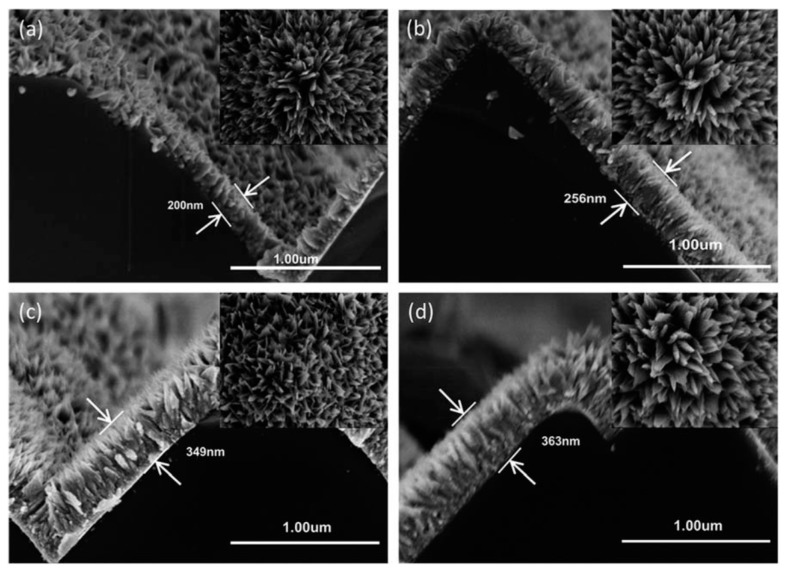
Cross-sectional field emission scanning electron microscope image of CuO NL grown on pyramid-textured Si substrates for (**a**) 30 min; (**b**) 45 min; (**c**) 60 min; and (**d**) 90 min in a two-step process. Inset of each image shows the corresponding top view. Reproduced in part from *J, Mater. Chem. A*, 2014, **2**, 6796 with permission from The Royal Society of Chemistry [47].

Another copper oxide nanostructure is the nanoleaf (NL). CuO NL can be solution synthesized by a two-step process on crystalline Si subtrates. In [47], CuO NLs were grown on commercial surface textured n^+^-p Si solar cells without passivation layer. After growth, the NLs have an average length of ~280 nm and width of ~83 nm (Figure 13). The NL layer enhances light absorption in the Si solar cell by acting as a graded index layer, which increases light scattering and trapping across a broad spectral region. In addition, the small CuO/n^+^Si heterojunction beneath each NL facilitates charge extraction from the device. When the morphology of the NL is optimized, the η of CuO NL/n^+^-p Si solar cell can reach 11.07%, which is significantly higher than the control device (9.39%) [47]. Very recently, CuO nanoparticles prepared by green synthesis have also been used as counter electrodes of dye sensitized solar cells [48].

In recent years, the photovoltaic behavior of CuO/fullerene (C_60_) heterojunctions has also been investigated. This inorganic-organic heterojunction structure is motivated by the fact that CuO is a p-type material while fullerene is an n-type (acceptor) material used for both heterojunction and bulk heterojunction organic solar cells. Oku *et al.* used electrochemical deposition to deposit CuO onto ITO working electrode by using an aqueous electrolyte consisting of CuSO_4_ and 1-lactic acid at 65 °C [49]. The C_60_ powder and top Al electrode were deposited on to CuO by vacuum evaporation. The measured η of the device, however, was extremely low (~10^−4^%) due to low *V*_oc_, FF and *J*_sc_. 

In summary, the key developments in CuO devices are the RF sputter deposition of both p-type CuO and n-type CuO*_x_*. Both oxides form heterojunctions with Si, but DAL is needed for CuO*_x_* and control of the interfacial layer is critical. The performance of CuO devices can be enhanced by the incorporation of CuO NP and NL to enhance light absorption.

## 5. Cu_2_O/CuO Thin Film Heterojunction Solar Cells 

Since CuO has a narrower energy band gap than Cu_2_O, a heterojunction comprising these two oxides of Cu can be formed from just two chemical elements. One advantage of the CuO/Cu_2_O heterojunction is that the refractive indices of Cu_2_O and CuO are quite similar, and, as a result, the reflectance of the CuO/Cu_2_O interface is relatively small and thus can be beneficial to solar cell performance [50]. In 1986, Siripala *et al.* used an electrochemical deposition technique to prepare n-Cu_2_O thin films by using a slightly acidic aqueous electrolyte during the deposition [51]. This technique was adapted by Wijesundera to fabricate p-CuO/n-Cu_2_O heterojunction PV devices [52]. In this study, a layer of Cu_2_O was first deposited at potentiostatic conditions at 55 °C using an electrolyte comprising copper (II) acetate monohydrate and sodium acetate. This layer was then completely oxidized to p-CuO by heating in air at 500 °C. A second layer of Cu_2_O was deposited by the same electrolyte at a higher bias potential to complete the formation of the heterojunction. 

Very recently, Jayathilaka *et al.* demonstrated a simple electrodeposition technique to fabricate p-CuO/n-Cu_2_O heterojunction solar cells [50]. The first p-CuO layer was deposited onto a Ti substrate at potentiostatic condition in a three electrode electrochemical cell using an alkaline electrolyte comprising lactic acid, copper (II) sulphate and sodium hydroxide. After post-deposition thermal annealing, the second layer of Cu_2_O was deposited at potentiostatic condition using a mixture of sodium acetate and copper (II) acetate. The conductivity types of the two electrodeposited layers were determined by the photocurrent response of a photoelectrochemical cell (PEC) using the Cu_2_O and CuO layers as a photocathode [50]. For the CuO photocathode, a negative spectral photocurrent shows that the CuO has p-type conductivity. A positive spectral photocurrent for Cu_2_O photocathode shows that the Cu_2_O has n-type conductivity. When the CuO/Cu_2_O heterojunction was used as photocathode, the spectral photocurrent exhibits opposite signs at different spectral regions. This shows that a p-CuO/n-Cu_2_O heterojunction has formed with longer wavelength radiation penetrating deeper into the junction. After surface defect passivation treatment of the Cu_2_O layer by ammounium sulphide (NH_4_)_2_S [50], a Ti/p-CuO/n-Cu_2_O/Au solar cell based on this heterojunction has a measured *V*_oc_ = 0.19 V, *J*_sc_ = 6.4 mA/cm^2^ and η = 0.52%. The η increased further to 0.64% after annealing at 100 °C for 45 min [50].

## 6. Cu_4_O_3_ Thin Film Heterojunction Solar cells

Thus far, there has only been one report on the application of Cu_4_O_3_ in thin film heterojunction solar cells. In [1], Meyer *et al.* reported preliminary results on a Cu_4_O_3_/GaN solar cell that was fabricated on double polished sapphire substrate. The n-type GaN was grown on sapphire and the heterojunction was formed by RF sputtering of Cu_4_O_3_ onto GaN. The combined absorber thickness was ~600 nm. Under AM 1.5G illumination, the heterojunction device showed a *J*_sc_ = 0.15 mA/cm^2^, *V*_oc_ = 0.87 V, *FF* = 67% and η = 0.009%. All photovoltaic parameters are lower than those of a Cu_2_O/GaN heterojunction solar cell fabricated for comparison.

Environmentally stable and efficient solar cells are crucial to meet the future demand for rewnewable energy. Thus, materials selection and suitable fabrication methods are essential. Earth abundant low cost material such as metal oxides and nitrides, sulphide based compounds and thin film silicon are very important [53,54,55,56,57,58,59]. As the copper oxides provide the suitable band gaps for single junction and tandem solar cells, it is worthwhile to find n-type materials with suitable band gap. Moreover, the sputter deposition technique is industry compatible and suitable for large scale deployment [60,61,62,63,64]. 

## 7. Conclusions

In this paper, the technological development of heterojunction solar cells based on Cu_2_O, CuO and Cu_4_O_3_ has been comprehensively reviewed. Amongst these three oxides of Cu, Cu_2_O devices are at the most advanced stage of development with the highest reported η. The rapid improvement in the past few years has been due to the use of low damage deposition processes for n-type semiconductors, improved band alignment and improved crystallinity of the junction materials. The main outsanding problems that merit further research are the passivation of electronic defects at the interface between Cu_2_O and the n-type semiconductor. The studies by Lee *et al.* using ALD buffer layers can be viewed as a first step in this direction [34]. The nature, energy distribution and mechanism of recombination at these interface states are not well understood at present. A more fundamental understanding of these defects should explain why the η of state of the art Cu_2_O devices are still well short of the theoretical maximum efficiency of 20% [6]. Deposition of semiconductors over a large area of Cu_2_O is another challenge that requires further technological development. 

For CuO heterojunction solar cells, the best reported η is still much lower than Cu_2_O devices. In order for further progress to be made, the basic material properties such as crystal structure, electronic structure, optical absorption and electronic properties will need to be determined. As with Cu_2_O, the control of the interface properties is crucial. Specifically, a low damage deposition process for CuO deposition on n-Si must be developed to avoid the formation of a Cu rich CuO*_x_* layer. A deposition process called successive ion layer adsorption and reaction (SILAR) that can deposit Cu_2_O and CuO at mild solution process conditions without ion bombardment is especially promising [65]. The recent observation of n-type behavior in CuO*_x_* suggests that CuO/CuO*_x_* junctions should be fabricated by sputtering and investigated for homojunction behavior [46].

Given the current stage of technological development of copper oxide heterojunction solar cells, a plausible near-term application of these earth abundant PV materials may be the mutli-junction, multi-terminal solar cells [14]. These devices consist of silicon cells connected in parallel with heterojunction solar cells based on other semiconductor materials. Unlike conventional tandem cells, each cell can be individually optimized in device design. The wider band gap of both Cu_2_O and CuO relative to Si can enhance the performance of Si cells, which are already near their theoretical limits by absorbing shorter wavelength photons. This critical review of the recent literature on copper oxide heterojunction solar cells has shown that, far from being an obsolete material system, the copper oxides are a versatile, sustainable PV material of growing interest and importance. 

## Figures and Tables

**Table 1:** Photovoltaic parameters of Cu_2_O and CuO heterojunction solar cells.

Heterojunction (Deposition Occurs on First Named Semiconductor)	Deposition Method	Open-Circuit Voltage *V*_oc_ (mV)	Short Circuit Current Density *J*_sc_ (mA/cm^2^)	Fill Factor *FF*	η (%) at (AM)	Ref.
Cu_2_O/ZnO	rf sputtering	342	2.29	0.3	0.14 (–)	[23]
Cu_2_O/ZnO:Al	PLD	400	7.1	0.4	1.2 (2)	[26]
Cu_2_O/ZnO	Ion beam sputtering	595	6.78	0.5	2 (1.5G)	[29]
Cu_2_O/ZnO	PLD	690	–	0.55	3.85 (1.5G)	[30]
Cu_2_O/Ga_2_O_3_/ZnO:Al	PLD	800	9.99	0.67	5.38 (1.5G)	[32]
Cu_2_O:Na/Al*_x_*Ga_1-*x*_O/ ZnO:Al (*x* = 0.025)	PLD	840	10.95	0.66	6.1 (1.5G)	[34]
Cu_2_O/a-ZTO/ZnO:Al	Atomic layer deposition	553	7.37	0.65	2.65 (1.5G)	[35]
n-Si/CuO	rf sputtering	330	6.27	0.2	0.41 (1.5D)	[39]
n-Si/CuO:N	rf sputtering	494	6.4	0.32	1.0 (1.5)	[41]
p-μc-Si:H/i-a-Si:H/n-CuO_x_	Plasma, reactive sputtering	495	13.68	0.449	3.04 (1.5)	[46]
ZnO/CuO/copper oxide nanopowder	PLD, hydrothermal	400	20.9	0.343	2.88 (1.5)	[20]
CuO/Cu_2_O	Electrochemical	220	6.8	–	0.64 (1.5)	[50]
GaN/Cu_4_O_3_	rf sputtering	870	0.15	0.67	0.009 (1.5 G)	[1]

D: direct.

## References

[1] Meyer B.K., Polity A., Reppin D., Becker M., Hering P., Klar P.J., Sander T.H., Reindl C., Benz J., Eickhoff M. (2012). Binary copper oxide semiconductors: From materials towards devices. Phys. Status Solidi B.

[2] Riordan M., Hoddeson L. (1997). Crystal Fire.

[3] Brattain W.H. (1951). The copper oxide rectifier. Rev. Mod. Phys..

[4] Drobny V.F., Pulfrey D.L. (1979). Properties of reactively-sputtered copper oxide thin films. Thin Solid Films.

[5] Olsen L.C., Addis F.W., Miller W. (1982). Experimental and theoretical studies of Cu_2_O solar cells. Sol. Cells.

[6] Olsen L.C., Bohara R.C., Urie M.W. (1979). Explanation for low-efficiency Cu_2_O Schottky-barrier solara cells. Appl. Phys. Lett..

[7] Rakhshani A.E. (1986). Preparation, characterization and photovoltaic properties of cuprous oxide—A review. Solid-State Electr..

[8] Scopus. http://www.scopus.com.

[9] Green M.A., Emery K., Hishikawa Y., Warta W., Dunlop E.D. (2016). Solar cell efficiency tables (version 47). Prog. Photovolt. Res. Appl..

[10] Dittrich T. (2014). Materials Concepts for Solar Cells.

[11] Nelson J. (2005). The Physics of Solar Cells.

[12] Fonash S. (2010). Solar Cell Device Physics.

[13] Gupta N., Singh R., Wu F., Narayan J., McMillen C., Alapatt G.F., Poole K.F., Hwu S.J., Sulejmanovic D., Young M. (2013). Deposition and characterization of nanostructured Cu_2_O thin-film for potential photovoltaic applications. J. Mater. Res..

[14] Singh R., Alapatt G.F., Kakhtakia A. (2013). Making solar cells a reality in every home: Opportunities and challenges for photovoltaic device design. IEEE J. Electron Dev. Soc..

[15] Wadia C., Alivisatos A.P., Kammen D.M. (2009). Materials availability expands the opportunity for large-scale photovoltaics deployment. Environ. Sci. Technol..

[16] Lee Y.S., Berfoni M., Chan M.K., Ceder G., Buonassisi T. Earth abundant materials for high efficiency heterojunction thin film solar cells. Proceedings of the 34th IEEE Photovoltaic Specialists Conference.

[17] Barquinha P., Martins R., Pereira L., Fortunato E. (2012). Transparent Oxide Electronics: From Materials to Devices.

[18] Sze S.M. (1981). Physics of Semiconductor Devices.

[19] Roosbroeck W.V., Shockley W. (1954). Photon-radiative recombination of electrons and holes in germanium. Phys. Rev..

[20] Bhuamik A., Haque A., Karnati P., Taufique M.F.N., Patel R., Ghosh K. (2014). Copper oxide based nanostructures for improved solar cell efficiency. Thin Solid Films.

[21] De Los Santos Valladares L., Hurtado Salinas D., Bustamante Dominguez A., Acosta Najarro D., Khondaker S.J., Mitrelias T., Barnes C.H.W., Albino Aguiar J., Majima Y. (2012). Crystallization and electrical resistivity of Cu_2_O and CuO obtained by thermal oxidation of Cu thin films on SiO_2_/Si substrates. Thin Solid Films.

[22] Biccari F. (2010). Defects and doping in Cu_2_O. Ph.D. Thesis.

[23] Herion J., Niekisch E.A., Scharl G. (1980). Investigation of metal oxide/cuprous oxide heterojunction solar cells. Sol. Energy Mater..

[24] Akimoto K., Ishizuka S., Yanagita M., Nawa Y., Paul G.K., Sakurai T. (2006). Thin film deposition of Cu_2_O and applications for solar cells. Sol. Energy.

[25] Minami T., Tanaka H., Shimakawa T., Miyata T., Sato H. (2004). High efficiency oxide heterojunction solar cells using Cu_2_O sheets. Jpn. J. Appl. Phys..

[26] Tanaka H., Shimakawa T., Miyata T., Sato H., Minami T. (2004). Electrical and optical properties of TCO-Cu_2_O heterojunction devices. Thin Solid Films.

[27] Minami T., Miyata T., Ihara K., Minamino Y., Tsukada S. (2006). Effect of ZnO film deposition methods on the photovoltaic properties of ZnO-Cu_2_O heterojunction devices. Thin Solid Films.

[28] Minami T., Ida S., Miyata T. (2002). High rate deposition of transparent conducting oxide thin films by vacuum arc plasma evaporation. Thin Solid Films.

[29] Mittiga A., Salza E., Sarto F., Tucci M., Vasanthi R. (2006). Heterojunction solar cell with 2% efficiency based on a Cu_2_O substrate. Appl. Phys. Lett..

[30] Minami T., Nishi Y., Miyata T., Nomoto J-L. (2011). High-efficiency oxide solar cells with ZnO/Cu_2_O heterojunction fabricated on thermally oxidized Cu_2_O sheets. Appl. Phys. Exp..

[31] Nishi Y., Miyata T., Minami T. (2012). Effect of inserting a thin buffer layer on the efficiency in n-ZnO/p-Cu_2_O heterojunction solar cells. J. Vac. Sci. Technol. A.

[32] Minami T., Nishi Y., Miyata T. (2013). High efficiency Cu_2_O-based heterojunction solar cells fabricated using a Ga_2_O_3_ thin film as N-type layer. Appl. Phys. Exp..

[33] Jacobi K., Zwicker G., Gutmann A. (1984). Work function, electron affinity and band bending of zinc oxide surfaces. Surf. Sci..

[34] Minami T., Nishi Y., Miyata T. (2015). Heterojunction solar cell with 6% efficiency based on an n-type aluminum-gallium-oxide thin film and p-type sodium-doped Cu_2_O sheet. Appl. Phys. Exp..

[35] Lee Y.S., Heo J., Siah S.C., Mailoa J.P., Brandt R.E., Kim S.B., Gordon R.G., Buonassisi T. (2013). Ultrathin amorphous zinc-tin-oxide buffer layer for enhancing heterojunction interface quality in metal-oxide solar cells. Energy Environ. Sci..

[36] Lee S.W., Lee Y.S., Heo J., Siah S.C., Chua D., Brandt R.E., Kim S.B., Mailoa J.P., Buonassisi T., Gordon R.G. (2014). Improved Cu_2_O-based solar cells using atomic layer deposition to control the Cu oxidation state at the p-n junction. Adv. Energy Mater..

[37] Lee Y.S., Heo J., Winkler M.T., Siah S.C., Kim S.B., Gordon R.G., Buonassisi T. (2013). Nitrogen-doped cuprous oxide as a p-type hole-transporting layer in thin-film solar cells. J. Mater. Chem. A.

[38] Zuo C., Ding L. (2015). Solution-processed Cu_2_O and CuO as hole transporting materials for efficient perovskite solar cells. Small.

[39] Gao F., Liu X.J., Zhang J.S., Song M.Z., Li N. (2012). Photovoltaic properties of the p-CuO/n-Si heterojunction prepared through reactive magnetron sputtering. J. Appl. Phys..

[40] Kumar V., Masudy-Panah S., Tan C.C., Wong T.K.S., Chi D.Z., Dalapati G.K. Copper oxide based low cost thin film solar cells. Proceedings of the IEEE International Nanoelectronics Conference.

[41] Masudy-Panah S., Dalapati G.K., Radhakrishnan K., Kumar A., Tan H.R., Kumar E.N., Vijila C., Tan C.C., Chi D.Z. (2015). p-CuO/n-Si heterojunction solar cells with high open circuit voltage and photocurrent through interfacial engineering. Prog. Photovolt. Res. Appl..

[42] Masudy-Panah S., Dalapati G.K., Radhakrishnan K., Kumar A., Tan H.R. (2014). Reduction of Cu-rich interfacial layer and improvement of bulk CuO property through two-step sputtering for p-CuO/n-Si heterojunction solar cell. J. Appl. Phys..

[43] Masudy-Panah S., Radhakrishnan K., Kumar A., Wong T.I., Yi R., Dalapati G.K. (2015). Optical band gap widening and phase transformation of nitrogen doped cupric oxide. J. Appl. Phys..

[44] Masudy-Panah S., Radhakrishnan K., Tan H.R., Yi R., Wong T.I., Dalapati G.K. (2015). Titanium doped cupric oxide for photovoltaic application. Sol. Energy Mater. Sol. Cells.

[45] Dalapati G.K., Kajen R.S., Masudy-Panah S., Sonar P. (2015). Defect analysis of sputter grown cupric oxide for optical and electronics application. J. Phys. D Appl. Phys..

[46] Lee S.H., Shin M., Yun S.J., Lim J.W. (2015). CuO_x_/a-Si:H heterojunction thin-film solar cell with an n-type μc-Si:H depletion-assisting layer. Prog. Photovolt. Res. Appl..

[47] Xia Y., Pu X., Liu J., Liang J., Liu P., Li X., Yu X. (2014). CuO nanoleaves enhance the c-Si solar cell efficiency. J. Mater. Chem. A.

[48] Sharma J.K., Akhtar M.S., Ameen S., Srivastava P., Singh G. (2015). Green synthesis of CuO nanoparticles with leaf extract of *Calotropis gigantea* and its dye-sensitized solar cells applications. J. Alloys Comp..

[49] Oku T., Motoyoshi R., Fujimoto K., Akiyama T., Jeyadevan B., Cuya J. (2011). Structures and photovoltaic properties of copper oxide/fullerene solar cells. J. Phys. Chem. Solids.

[50] Jayathilaka C., Kapaklis V., Siripala W., Jayanetti S. (2015). Improved efficiency of electrodeposited p-CuO/n-Cu_2_O heterojunction solar cells. Appl. Phys. Exp..

[51] Siripala W., Jayakody J.R.P. (1986). Observation of n-type photoconducitivty in electrodeposited copper oxide film electrodes in a photoelectrochemical cell. Sol. Energy Mater..

[52] Wijesdundera R.P. (2010). Fabrication of the CuO/Cu_2_O heterojunction using an electrodeposition technique for solar cell applications. Semicond. Sci. Technol..

[53] Yang M., Zakutayev A., Vidal J., Zhang X., Ginley D.S., DiSalvo F.J. (2013). Strong optical absorption in CuTaN_2_ nitride delafossite. Energy Environ. Sci..

[54] Katagiri H., Saitoh K., Washio T., Shinohara H., Kurumadani T., Miyajima S. (2001). Development of thin film solar cell based on Cu_2_ZnSnS_4_ thin films. Sol. Energy Mater. Sol. Cells.

[55] Katagiri H., Jimbo K., Yamada S., Kamimura T., Maw W.S., Fukano T., Ito T., Motohiro T. (2008). Enhanced conversion efficiencies of Cu_2_ZnSnS_4_-based thin film solar cells. Appl. Phys. Exp..

[56] Müller J., Recha B., Springerb J., Vanecekb M. (2004). TCO and light trapping in silicon thin film solar cells. Sol. Energy.

[57] Masudy-Panah S., Kumar V., Tan C.C., Radhaknshnan K., Chi D.Z. Impact of metal contact on the performance of cupric oxide based thin film solar cells. Proceedings of the IEEE 5th International Nanoelectronics Conference.

[58] Dalapati G.K., Masudy-Panah S., Kumar A., Tan C.C., Tan H.R., Chi D. (2015). Aluminium alloyed iron-silicide/silicon solar cells: A simple approach for low cost environmental-friendly photovoltaic technology. Sci. Rep..

[59] Dalapati G.K., Batabyal S.K., Masudy-Panah S., Su Z., Kushwaha A., Wong T.I., Liu H.F., Bhat T., Iskander A., Lim Y.F. (2015). Sputter grown sub-micrometer thick Cu_2_ZnSnS_4_ thin film for photovoltaic device application. Mater. Lett..

[60] Dalapati G.K., Liew S.L., Wong A.S.W., Chai Y., Chiam S.Y., Chi D.Z. (2011). Photovoltaic characteristics of p-β -FeSi2(Al)/n-Si(100) heterojunction solar cells and the effects of interfacial engineering. Appl. Phys. Lett..

[61] Dalapati G.K., Kumar A., Tan C.C., Liew S.L., Sonar P., Seng H.L., Hui H.K., Tripathi S., Chi D.Z. (2013). Impact of Al Passivation and cosputter on the structural property of β-FeSi2 for Al-Doped β -FeSi2/n-Si(100) based solar cells application. ACS Appl. Mater. Interfaces.

[62] Tan C.C., Dalapati G.K., Tan H.R., Bosman M., Hui H.K., Tripathy S., Chi D.Z. (2015). Crystallization of sputter-deposited amorphous (FeSi2)_1–*x*_Al*_x_* thin films. Cryst. Growth Des..

[63] Masudy-Panah S., Moakhar R.S., Chua C.S., Hui R.T., Wong T.I., Chi D.Z., Dalapati G.K. (2016). Nanocrystal Engineering of Sputter-Grown CuO Photocathode for Visible-Light-Driven Electrochemical Water Splitting. ACS Appl. Mater. Interfaces.

[64] Dalapati G.K., Masudy-Panah S., Chua S.T., Sharma M., Wong T.I., Tan H.R., Chi D.Z. (2016). Color tunable low cost transparent heat reflector using copper and titanium oxide for energy saving application. Sci. Rep..

[65] Chatterjee S., Saha S.K., Pal A.J. (2016). Formation of all-oxide solar cells in atmospheric condition based on Cu_2_O thin-films grown through SILAR technique. Sol. Energy Mater. Sol. Cells.

